# Examining the association between diet-related situational factor and dietary behavior: an observational study of diet-related situational factors in stroke patients during rehabilitation

**DOI:** 10.3389/fnut.2025.1696883

**Published:** 2025-11-12

**Authors:** Weiying Zhong, Xi Pan, Jiaxuan Li, Yi Zhang, Lei Chen, Xueqi Sun, Zhi Wang, Lan Xu

**Affiliations:** 1 Department of Nursing, The First Affiliated Hospital of Soochow University, Suzhou, Jiangsu, China; 2 Department of Neurology, The First Affiliated Hospital of Soochow University, Suzhou, Jiangsu, China

**Keywords:** stroke, rehabilitation, dietary behavior, situational factor, meal context

## Abstract

**Background:**

Dietary behavior is affected by various factors and adverse dietary behavior is a risk factor for stroke recurrence. The current study examined the relationship between dietary behavior and relevant situational factors in stroke patients during rehabilitation.

**Methods:**

A total of 257 stroke patients recorded dietary intake and assessed diet-related situational factors at each meal via an information platform for three consecutive days during rehabilitation. A multiple logistic model was developed to analyze how diet-related situational factors influence dietary behavior.

**Results:**

A total of 183 participants completed the study. Lunch accounted for the highest proportion (44.5%) of energy-qualified meals and breakfast for the lowest proportion (37.8%). Patients with a noisy dining environment, who needed help from others to cook or shop for groceries independently or who ate in a public open space were more likely to have adverse dietary behavior at breakfast. Patients who had a noisy dining environment, who needed help from others to cook or shop for groceries or who ate with friends were more likely to have adverse dietary behavior at lunch. Patients in the early stages of recovery, who had a noisy dining environment, who needed help from others to cook or shop for groceries, who ate with friends and had a high level of satisfaction with eating were more likely to show adverse dietary behavior at dinner.

**Conclusion:**

Poor dietary behavior was common in stroke patients with low probability of qualified energy intake during rehabilitation. Meal location and companions were among the situational factors that influenced dietary behavior.

## 1 Introduction

Stroke is one of the leading causes of disability and mortality worldwide. Its high recurrence rate aggravates brain injury, leading to impaired quality of life, elevated risks of disability and death, and a considerable economic and social burden on healthcare systems (1–3). Stroke patients suffer a shorter time of intravascular plaque stabilization during rehabilitation and the continuing instability of the plaque increases recurrence risk. Risk factors include those that are non-modifiable (1), such as gender, age, genetics and family history (1–5), and those for which medical interventions may be available, such as hypertension, diabetes mellitus, dyslipidemia, atrial fibrillation and carotid artery disease (6). A third category of risk factors includes those that are modifiable by the individual, such as adverse dietary behavior, insufficient physical activity, smoking, alcohol abuse, obesity and other lifestyle factors. Control of modifiable risk factors may make a substantial contribution to reducing the risk of stroke recurrence during rehabilitation and lifestyle intervention is considered a cost-effective strategy (7). Guidelines for secondary prevention of stroke suggest that changing adverse dietary behavior may prevent stroke recurrence, (8–10) by influencing pathophysiological mechanisms and altering patients’ clinical outcomes. Healthy dietary habits promote weight management, lower blood pressure, reduce inflammatory markers and improve blood lipid profiles, leading to enhanced cardiovascular metabolic health and reducing stroke incidence and mortality.

Dietary behavior is highly complex (11) and influenced by many interacting factors (12). The assessment of dietary behavior in a laboratory setting or via retrospective measures presents a challenge due to the transient nature and dependence on immediate environmental contexts (13). Laboratory settings are difficult to replicate real-life dietary contexts, while retrospective measurements often introduce bias due to the transient nature of dietary behavior and delays in recording. Compared with retrospective questionnaires, internet-based dietary recording methods significantly reduce the time gap between consumption and recording (14, 15). This not only minimizes recall bias but also improves the accuracy and reliability of dietary intake data. For stroke patients, whose memory and cognitive functions may be impaired, such timely recording methods are particularly valuable for capturing real-life dietary behaviors and enabling more precise assessments of dietary intake.

Dietary behavior encompasses dietary patterns and situational factors, situational factors are defined as temporal and spatial situation-specific variables, (16) such as meal location and companions. To comprehensively understand and effectively modify dietary behavior, situational factors must be assessed in real-world scenarios to recapitulate the natural pattern of eating behavior and reveal the psychological, physiological and behavioral mechanisms responsible. Dietary choices are acknowledged to be strongly influenced by where, with whom and in what environmental context one eats. Chwyl et al. (17) demonstrated that overweight individuals were at elevated risk of binge eating during social activities but not while eating at school/work, performing household tasks, praying or meditating. Both the type and degree of environmental support given to stroke patients during meals have complex effects on eating behavior. Qualitative studies exploring post-stroke eating behavior indicated that mobility limitations and negative emotional states may cause restrictive eating patterns (18) which may be mitigated by improved environmental support. Diet-related situational factors have been investigated in different populations. For example, an 8-week dietary intervention during which hypertensive patients were geotagged and received an intervention message via an app when they entered a restaurant or grocery store was conducted by Dorsch et al. (19, 20) and showed reduced sodium intake in the app intervention group. Identifying and analyzing situational factors may facilitate similar dietary interventions in stroke patients.

Participants in dietary surveys must have a sufficient level of numeracy and literacy to weigh or estimate food consumption (21) and estimates remain relatively inaccurate, leading to error. Middle-aged or elderly stroke victims may have memory loss, neurological or cognitive impairment due to ischemic or hemorrhagic brain damage (22) which compounds the difficulties of dietary surveys. The Food Estimation Imaging Method (FEIM) is a food quantity estimation tool that uses visual reference to overcome the shortcomings of traditional dietary surveys. A food atlas was incorporated as an auxiliary FEIM tool to aid self-reporting among the participants of the current study in Supplementary Figure 1. The food atlas allows comparison of food or portion size, background scale coordinates and familiar objects in daily life via three visual reference components to overcome imaging errors which may obscure the accuracy of food quantity estimates. The atlas has utility for stroke patients during rehabilitation. Our research team has developed a “Stroke Health Behavior Management Platform” to facilitate the monitoring and management of post-stroke health behaviors and enable internet-based dietary recording. When integrated with FEIM, this platform allows for precise dietary assessment, enhancing the accuracy and reliability of data in the present study.

This research focuses on the study of situational factors, emphasizing real-time, naturalistic context and ecological validity. Centered on authentic rehabilitation scenarios, it integrates precise dietary assessment tools with real-time situational recording to systematically investigate diet-related situational factors and to examine the dietary behaviors of stroke patients during rehabilitation. Stroke patients assessed dietary intake using FEIM and recorded situational factors, such as location (eating at home or elsewhere), social context (eating alone or company) and satisfaction with food, in real time during the current study. This study examines the relationship between situational factors and dietary behavior in stroke patients during rehabilitation, with the aim of providing empirical evidence to inform the formulation of situation-specific dietary interventions, ultimately facilitating the reduction of stroke recurrence and the enhancement of rehabilitation outcomes.

## 2 Materials and methods

### 2.1 Study design and population

A cross-sectional survey of dietary intake and situational factors was conducted in stroke patients during rehabilitation over three consecutive days between March 2024 and December 2024. Stroke patients visiting the Department of Neurology, First Affiliated Hospital of Soochow University during rehabilitation between March 2024 and December 2024 were recruited.

Inclusion criteria: patients who confirmed diagnosis of stroke, with clinical manifestations consistent with the Chinese diagnostic criteria for stroke; (23) stroke patients during rehabilitation, diagnosed 2 weeks to 6 months after the onset of stroke; (24) either the participants themselves or their caregivers possessed a smartphone and were capable of operating WeChat for real-time recording; provided informed consent and voluntarily agreed to participate.

Exclusion criteria: potential participants were excluded if they met any of the following criteria: suffering from advanced liver, pulmonary, renal, or cardiac disease that could interfere with participation or dietary behavior; having active, pre-existing major neurological diseases or psychiatric conditions (e.g., major depression, bipolar disorder, schizophrenia, or dementia) that affected their ability to participate or accurately record information; having contraindications for participation due to therapeutic or other medical reasons, as determined by the attending physician; concurrently participating in other studies on similar topics to avoid potential confounding effects; being unable or unwilling to comply with study procedures.

A total of 289 stroke patients were identified, of whom 257 agreed to participate. Patients completed a general information questionnaire covering sociodemographic and disease-related information before the start of the study. Dietary intake and situational factors were self-recorded for three consecutive days using the dietary survey information platform described above. The 3-day assessment duration was chosen based on previous studies. Ethical approval was granted by the Medical Ethics Research Committee, First Affiliated Hospital of Soochow University (Approval No. 2024012).

### 2.2 Patient characteristics

A questionnaire was developed to collate sociodemographic and disease-related information, including age (years), sex, body mass index (BMI, kg/m^2^), occupation, history of smoking, history of alcohol consumption, educational attainment, marital status, place of residence, residential status, gross annual household income (10,000 yuan) and medical insurance. Height and weight were measured by researchers and BMI was calculated. Other information was self-reported.

Patients were divided into two groups depending on age: < 60 and ≥ 60 years. BMI was categorized as underweight (BMI < 18.5), normal weight (18.5 ≤ BMI < 25) or overweight/obese (BMI ≥ 25) (25). A history of smoking (smokers) was defined as smoking at least 1 cigarette per day for 1 year (26) and a history of alcohol consumption as drinking ≥ 20 g/d for more than 3 years (27). Educational attainment was categorized as elementary school and below, high school/secondary school or college and above. Marital status was categorized as married or unmarried/divorced/widowed. Place of residence was categorized as rural, town, county or urban. Residential status was categorized as living alone or living with family. Gross annual household income was defined as the sum of 1 year’s income for all household members and was categorized as < 1, 1–3, 4–8; 9–15, 16–30 or 31–100 (× 10,000 Yuan). Medical insurance was categorized as basic medical insurance for urban workers, basic medical insurance for urban and rural residents or self-funded medical insurance. Type and severity of stroke, staging of rehabilitation period, recovery of physical function, ability to perform activities of daily living (ADL) and swallowing function were accessed from medical records.

Patients were divided into three groups depending on stroke type: ischemic stroke, hemorrhagic stroke or ischemic combined with hemorrhagic stroke. Rehabilitation was divided into initial (1–8 weeks post-stroke) and ongoing (8 weeks–6 months post-stroke) periods, according to the criteria proposed by Kirkevold (24). Stroke severity was measured using the National Institute of Health Stroke Scale (NIHSS) which ranges from 0 to 42 with higher scores indicating more severe strokes (28): mild: 0–4, moderate: 5–12, moderately severe: 16–20 and severe: > 20. Current participants were categorized into two groups mild (≤ 4) or non-mild (> 4). ADL was assessed by the Barthel Index (BI) which ranges from 0 to 100 with higher scores indicating greater independence in ADL: heavy dependence: < 40, moderate dependence: 40–60, mild dependence: 60–99 and no dependence: 100 (29). Current participants were categorized into two groups: moderately to severely dependent (≤ 60) or mildly to not dependent (> 60). Swallowing function was assessed by the Water Swallowing Test (WST) which measures the time taken to drink 30 ml water, number of swallows, hoarseness and choking giving a higher grade to worse function: grade 1: ≤ 5 s; grade 1–2 (suspicious): > 5 s; grades 3–5 (abnormal) (30). Current participants were categorized into three groups: normal, suspicious and abnormal.

### 2.3 Data collation platform

Atlas content was uploaded to the information platform in Supplementary Figure 2 and 3-day dietary intake and situational factors were collected by dietary survey through the information platform previously developed by the research group. Data included meal type, whether the meal was eaten or not, time of eating, food type, food portion and nutrients. Meal types included breakfast, lunch, dinner and extra meals. Food type and portion refer to standards given in the Dietary Guidelines for Chinese Residents 2022 in which food types include cereals and potatoes, vegetables, fruits, livestock and poultry, meat, eggs, fish and shrimp, legumes and products and milk and products. Food portion was measured in grams and nutrients were recorded. For example, 100 g steamed bread contains 223 kcal, 47.00 g carbohydrates and 7.00 g protein. Participants accessed the information platform via the WeChat mini program, where they received registration assistance and completed data entry. Daily energy requirements were automatically calculated from height, weight and caloric recommendations of the Dietary Guidelines for Stroke Patients. Energy intake per meal was calculated according to the 3:4:3 three-meal ratio recommended by the Chinese Resident’s Dietary Guidelines 2022. Each meal was automatically assessed for adequacy and data was uploaded. Participants entered situational factors prior to each meal, such as meal location, companions, intention to eat, need for help during the meal, meal satisfaction, negative emotions during the meal, ability to cook independently, ability to shop for groceries independently and environmental quietness during the meal.

### 2.4 Recording of dietary intake and situational factors

Participants were given instructions on the use of the information platform and data completion prior to the study onset and were reminded by phone call or WeChat message to fill in data or where data appeared doubtful or incomplete. Data was checked at 8 p.m. each evening to ensure completeness and the minimum data entry for analysis was dietary intake and situational factors for three meals per day: breakfast, lunch and dinner. All participants used the same information platform.

### 2.5 Statistical analyses

All statistical analyses were performed using SPSS 25.0. Descriptive data on patient characteristics were calculated for all variables. Categorical variables are expressed as frequency/percentage and compared using the two-sided chi-square test or Fisher exact test. Continuous data are reported as mean ± standard deviation (SD) or median [interquartile range (IQR)] and analyzed by independent *t*-test or Mann Whitney *U*-test. Continuous variables were dichotomized or categorized where necessary. The chi-square test and Fisher’s exact test were used to compare differences in baseline information between included and lost subjects. Correlation analysis was performed for the association between energy intake and situational factors. Univariate analysis was first performed to investigate the effect of situational factors on qualified energy intake. Then, Benjamini-Hochberg (BH)/Holm-Bonferroni (Holm) correction was incorporated to control for multiple comparisons. Finally, a multiple logistic model was established. A two-tailed *p*-value < 0.05 was considered to indicate statistical significance.

## 3 Results

### 3.1 Basic characteristics

A total of 257 recovering stroke patients who met the inclusion criteria and gave informed consent were recruited. 183 patients completed the study and 74 withdrew, giving a dropout rate of 28.8% (flowchart and reasons for refusal are presented in Supplementary Figure 3). A total of 159 (86.9%) participants were under 60 years; 135 (73.8%) were male and 24 (13.1%) were farmers in Supplementary Table 1. No significant differences in demographic characteristics were present between included and lost subjects.

### 3.2 Qualified energy intake in stroke patients during rehabilitation

A total of 1,640 meals were consumed by 183 stroke patients during the 3-day study, 543 for breakfast, 548 for lunch and 549 for dinner in Supplementary Table 2. Lunch accounted for the highest proportion of energy-qualified meals, with 44.5% (244 out of 548 meals) meeting the energy intake criteria, compared to 37.8% (205 out of 543 meals) for breakfast and 40.1% (220 out of 549 meals) for dinner.

### 3.3 Univariate correlates of diet-related situational factors and dietary behavior

Univariate analysis of demographic characteristics showed that gender, age, occupation, place of residence, history of alcohol consumption, stroke type and rehabilitation stage were significantly associated with qualified energy intake at breakfast; gender, age, occupation and rehabilitation stage at lunch and gender, stroke type, body mass index, rehabilitation stage and Barthel index at dinner in Supplementary Table 3.

Univariate analysis of situational factors showed meal location to be associated with qualified energy intake at breakfast and lunch, meal companions at lunch and dinner, the degree of needing help with meals at breakfast and lunch and the degree of meal satisfaction at dinner. Noise in the eating environment, the degree of needing help with independent cooking and the degree of needing help with independent grocery shopping were associated with qualified energy intake for all three meals in Supplementary Tables 4–8.

### 3.4 Multiple logistic model of diet-related situational factors and dietary behavior

Multiple logistic models were constructed using energy intake at each meal as the dependent variable and comparing variables that were statistically significant in the univariate analysis as independent variables.

#### 3.4.1 Breakfast

Gender, age, stroke type, history of alcohol consumption, rehabilitation stage, the degree of noise in the dining environment, the need for help to cook independently, the need for help to buy groceries independently and meal location all affected breakfast energy intake in Supplementary Table 9.

Personal characteristics: Male patients (*n* = 135) had a higher probability of insufficient energy intake and female patients (*n* = 48) of qualified energy intake (*p* = 0.003, OR = 0.471, 95% CI: 0.285∼0.777). Patients < 60 years (*n* = 159) had a higher probability of qualified energy intake and those > 60 years (*n* = 124) had a higher probability of excessive energy intake (*p* = 0.037, OR = 2.747, 95% CI: 1.063∼7.097). Patients with alcohol consumption history (*n* = 50) were more likely to achieve qualified energy intake, whereas non-drinkers showed higher probability of excessive intake (*p* = 0.010, OR = 0.387, 95% CI: 0.188∼0.796). Patients who had ischemic stroke (*n* = 177) had a lower probability of qualified energy intake (*p* = 0.044, OR = 7.216, 95% CI: 1.057∼49.270) (*p* = 0.030, OR = 9.768,95% CI: 1.241∼76.853). Patients had a higher probability of qualified energy intake during initial rehabilitation (*n* = 62) than during ongoing rehabilitation (*n* = 121) (*p* < 0.001, OR = 3.170, 95% CI: 1.754∼5.728).

Situational factors: Patients with a quieter dining environment (*p* = 0.001, OR = 2.696, 95% CI: 1.489∼4.884), less need for help with cooking independently (*p* = 0.001, OR = 2.704,95% CI: 1.500∼4.876) and less need for help with shopping for groceries independently (*p* < 0.001, OR = 3.411,95% CI: 1.867∼6.232) were more likely to have qualified energy intake and other patients were more likely to have excessive energy intake. Patients were more likely to have excessive energy intake in public open spaces (*p* = 0.035, OR = 2.055, 95% CI:1.132∼30.641).

#### 3.4.2 Lunch

Gender, degree of noise in the eating environment, need for help to cook independently, need for help to shop for groceries independently and meal companions all affected qualified energy intake at lunch in Supplementary Table 10.

Personal characteristics: Female patients (*n* = 48) had a higher probability of excessive energy intake and male patients (*n* = 135) of insufficient energy intake (*p* = 0.008, OR = 0.469, 95% CI: 0.268∼0.822).

Situational factors: Patients with a quieter dining environment (*p* < 0.001, OR = 2.672, 95% CI: 1.682∼4.245), less need for help with cooking independently (*p* < 0.001, OR = 2.682, 95% CI: 1.684∼4.272) and less need for help with buying food independently (*p* < 0.001, OR = 2.589, 95% CI: 1.633∼4.103) were more likely to have excessive energy intake. Patients who ate alone were more likely to have a qualified energy intake and intake was more likely to be insufficient if friends were present (*p* = 0.001, OR = 6.573, 95% CI: 2.161∼19.994).

#### 3.4.3 Dinner

Gender, rehabilitation stage, capacity for self-care, degree of noise in the dining environment, need for help with cooking independently, need for help with shopping for groceries independently, and degree of satisfaction with eating and meal companions all affected qualified energy intake at dinner in Supplementary Table 11.

Personal characteristics: Female patients (*n* = 48) had a higher probability of qualified energy intake and male patients (*n* = 135) of insufficient energy intake (*p* = 0.013, OR = 0.563, 95% CI: 0.357∼0.888). Patients in the initial rehabilitation (*n* = 62) were more likely to have an excessive energy intake and those in ongoing rehabilitation (*n* = 121) to have excessive energy intake (*p* < 0.001, OR = 0.347, 95% CI: 0.199∼0.602).

Situational factors: The presence of friends was associated with a greater probability of insufficient energy intake (*p* = 0.001, OR = 4.833, 95% CI: 1.921∼12.159) and excessive energy intake was associated with dinners taken alone or with family (*p* = 0.006, OR = 2.259, 95% CI: 1.259∼4.053). Patients with better self-care ability had a higher probability of excessive energy intake, whereas those with limited self-care ability were more likely to achieve qualified energy intake (*p* = 0.022, OR = 11.343, 95% CI: 1.409∼91.301). A noisy dining environment (*p* < 0.001, OR = 2.598, 95% CI: 1.533∼4.401), a need for help with cooking independently (*p* < 0.001, OR = 2.631, 95% CI: 1.551∼4.463) and a need for help with shopping for groceries independently were linked to a higher probability of excessive energy intake (*p* < 0.001, OR = 2.902, 95% CI: 1.707∼4.934). Insufficient energy intake was more likely to occur in patients with a higher level of eating satisfaction (*p* = 0.005, OR = 3.274, 95% CI: 1.434∼7.477).

## 4 Discussion

Only a small proportion of meals taken by patients in the current cohort had a qualified energy intake. Post-stroke swallowing dysfunction has an impact on normal eating and may affect choice of food type (31) with implications for energy intake. The current findings were consistent with those of a previous study conducted by Tang et al. (32) Many stroke patients also suffer loss of appetite (33) due to the nature of the disease, medication side effects or psychological factors that reduce interest in food, food intake and energy intake. Stroke patients generally have a low activity level during rehabilitation which may be below recommended levels and this also has an impact on energy needs and intake (34, 35). Thus, swallowing dysfunction, loss of appetite and reduced physical activity may all affect food and energy intake during post-stroke rehabilitation. Healthcare professionals may give nutritional support, psychological counseling and encourage increased physical activity to ensure appropriate energy intake and improve patients’ quality of life.

It has traditionally been considered that unhealthy dietary behavior is an individual choice. However, the current study highlights the impact of situational factors. Social environment affected energy intake with stroke patients more likely to achieve qualified energy intake when dining alone or with family and more prone to insufficient energy intake when other companions were present. Meal companions have previously been acknowledged to influence and regulate dietary behavior through social pressure (36, 37). The current findings were consistent with those of previous studies. Dietary behavior may be influenced by the dining environment and social interaction. Dining alone or with family represents a quiet, comfortable situation that allows a patient to focus more on the meal and adjust food intake according to energy needs. Frequent social interactions and a noisy environment distract the patient from their own dietary needs and foster errors in judging food intake. In addition, patients may focus on social etiquette and group atmosphere, neglecting dietary needs and increasing the risk of inadequate energy intake. Companions may affect dietary behavior and qualified energy intake by distracting the patient. Based on the findings of this study, meal companions should be selected according to the individual conditions of stroke patients in daily life. For those with insufficient energy intake, unnecessary social interactions during meals should be minimized. Establishing a quiet and comfortable dining environment, where the patient eats alone or with family members, can enhance focus on dietary needs and facilitate appropriate food intake. In addition, community and healthcare institutions should fully consider social and environmental factors when organizing group meals for stroke patients to promote adequate energy intake.

Post-stroke patients were more likely to have an excessive energy intake when eating in public places. Previous studies have indicated that food purchase location or social setting may influence food choice and small changes on numerous occasions may significantly affect overall diet quality over time (38, 39). Food choice variety on offer in public places tends to encourage over-consumption and a noisy environment is distracting, affecting the judgment of satiety. By contrast, patients who eat at home are more likely to achieve up-to-compliance intake and food preparation by the caregiver will influence intake (40). The results of the present study indicate that when dining in public settings, stroke patients should exercise greater self-regulation in their food choices and minimize the influence of environmental distractions to avoid overeating. Family members accompanying patients are advised to choose quiet restaurants with minimal distractions. Furthermore, the catering industry should consider designing supportive dining environments for stroke patients by providing portion-controlled menu options and maintaining a calm atmosphere to facilitate appropriate energy intake.

A higher meal satisfaction level was associated with greater likelihood of insufficient energy intake, perhaps related to psychological perceptions and dietary structure. Patients may focus on food taste and satisfaction, neglecting nutritional value and a satisfying eating experience may cause an illusory sense of fullness and end eating prematurely. Patients with lower levels of eating satisfaction may pay more attention to nutritional balance and energy intake, producing dietary behavior more in line with medical recommendations and satisfactory energy intake. Stroke patients and their families should develop a proper understanding of the relationship between dining satisfaction and nutritional intake, and should not neglect nutritional balance or energy requirements merely because of palatable food or a pleasurable dining experience. Patients are encouraged to focus proactively on the nutritional composition of their meals and optimize dietary structure while enjoying palatable food. When preparing meals, family members should prioritize both palatability and nutritional adequacy. Such practices can help guide patients to establish healthy dietary habits and prevent insufficient energy intake resulting from an excessive emphasis on dining satisfaction alone.

The capacity for independent cooking and shopping is a reflection of the patient’s self-management and this factor increased the probability of qualified energy intake (41). Dependence on others may weaken self-esteem, fostering doubts regarding the capacity for self-management and manifesting through limited food choices. Patients who are unable to dictate their own dietary behavior are susceptible to an unstable energy intake and independent cooking and shopping give greater autonomy. Involvement in ingredient selection and cooking may aid the perception of flavor, texture and satiety. Excessive dependence on others means that the focus shifts to interaction and communication with caregivers and away from food and energy intake. Independence in cooking and grocery shopping enhances self-management, self-confidence and motivation in disease recovery promoting a healthy dietary structure. Thus, the fostering of patients’ independence and self-management with attention to the psychological state and eating experience is likely to promote qualified energy intake. In terms of the capacity for independent cooking and shopping, future research could focus on developing and evaluating interventions designed to enhance patients’ self-management skills. These may include cooking programs specifically tailored for stroke patients, emphasizing both practical cooking techniques and nutritional balance. Furthermore, studies could investigate the role of community support in promoting independent living and improving dietary behavior and energy intake among stroke patients during rehabilitation.

Multidimensional situational factors may be considered in future studies to inform nutritional guidance programs. Stroke patients may be guided to adjust bad dietary behavior and improve health management.

In conclusion, the dietary behavior of post-stroke patients during rehabilitation was complex and represented a situation where qualified energy intake was often lacking. Situational factors, including meal companions, eating environment, environmental noise and the capacity for independent shopping and cooking, influenced dietary behavior and patients’ eating choices and habits. The findings of the current study may inform the development of targeted dietary interventions.

We acknowledge some limitations to the current study. A single hospital in China was involved which limits generalization to other populations. The current study was cross-sectional and longitudinal changes to dietary behavior and situational factors were not explored. The scope of the patient population and longitudinal factors may be considered in future work to expose trends of dietary behavioral changes and causal relationships. Moreover, by further exploring the potential implications outlined above, future research can build on the current findings to develop more effective and comprehensive strategies for improving the dietary behavior and health outcomes of stroke patients during rehabilitation.

## 5 Conclusion

The dietary behavior of post-stroke patients during rehabilitation was complex and represented a situation where qualified energy intake was often lacking. Situational factors, including meal companions, eating environment, and the capacity for independent shopping and cooking, influenced dietary behavior and patients’ eating choices and habits. The findings of the current study may inform the development of targeted dietary interventions.

## 6 Limitations and prospects

We acknowledge some limitations to the current study. A single hospital in China was involved which limits generalization to other populations. The current study was cross-sectional and longitudinal changes to dietary behavior and situational factors were not explored. The scope of the patient population and longitudinal factors may be considered to expose trends of dietary behavioral changes and causal relationships.

## Data Availability

The raw data supporting the conclusions of this article will be made available by the authors, without undue reservation.

## Supplementary material

Supplementary Figure 1 Food atlas.

Supplementary Figure 2 Information platform.

Supplementary Figure 3 Recruitment flowchart.
