# Supplementary material for: Examining the association between diet-related situational factor and dietary behavior: an observational study of diet-related situational factors in stroke patients during rehabilitation
Source: Front Nutr. 2025 Nov 12;12:1696883. doi: 10.3389/fnut.2025.1696883 (PMC12648219; doi:10.3389/fnut.2025.1696883)
Supplement: Supplementary file 2 [file Table_2.docx]

| **Table2-1** Qualified energy intake in stroke patients during rehabilitation period | | | | | | | | |
| --- | --- | --- | --- | --- | --- | --- | --- | --- |
| Energy intake | Breakfast | | Lunch | | Dinner | |  | |
|  | ***n*** | **%** | ***n*** | **%** | ***n*** | **%** | ***χ*^2^** | ***P*** |
| ***Insufficient*** | 252 | 46.4 | 148 | 27.0 | 232 | 42.3 | 57.423 | <0.001* |
| ***Qualified*** | 205 | 37.8 | 244 | 44.5 | 220 | 40.1 |  |  |
| ***Excessive*** | 86 | 15.8 | 156 | 28.5 | 97 | 17.7 |  |  |

| **Table2-2** Pairwise Comparison of Energy Intake Among Different Groups (Holm and BH Corrections) | | | |
| --- | --- | --- | --- |
| Comparison | ***Raw P*** | ***Adjusted P (Holm)*** | ***Adjusted p (BH)*** |
| ***Breakfast vs Lunch*** | <0.001* | <0.001* | <0.001* |
| ***Breakfast vs Dinner*** | <0.001* | <0.001* | <0.001* |
| ***Lunch vs Dinner*** | <0.001* | <0.001* | <0.001* |
