# Supplementary material for: Examining the association between diet-related situational factor and dietary behavior: an observational study of diet-related situational factors in stroke patients during rehabilitation
Source: Front Nutr. 2025 Nov 12;12:1696883. doi: 10.3389/fnut.2025.1696883 (PMC12648219; doi:10.3389/fnut.2025.1696883)
Supplement: Supplementary file 3 [file Table_3.docx]

| **Table3** The univariate model of the effects of sociodemographic and disease-related information on energy intake | | | | | | | | |
| --- | --- | --- | --- | --- | --- | --- | --- | --- |
| Characteristic | ***N*** | ***%*** | Breakfast | | Lunch | | Dinner | |
|  |  |  | *H/****χ*^2^** | *P* | *H/****χ*^2^** | *P* | *H/****χ*^2^** | *P* |
| ***Gender*** |  |  | 7.852 | 0.020 | 7.355 | 0.025 | 8.015 | 0.018 |
| Male | 135 | 73.8 |  |  |  |  |  |  |
| Female | 48 | 26.2 |  |  |  |  |  |  |
| ***Age(years)*** |  |  | 22.404 | ＜0.001 | 11.217 | 0.004 | 1.577 | 0.455 |
| <60 | 159 | 86.9 |  |  |  |  |  |  |
| ≥60 | 24 | 13.1 |  |  |  |  |  |  |
| ***Occupation*** |  |  | 22.735 | 0.012 | 27.937 | 0.002 | 7.094 | 0.717 |
| Farmer | 24 | 13.1 |  |  |  |  |  |  |
| Workers and technical operators | 69 | 37.7 |  |  |  |  |  |  |
| Government and public institutions personnel | 13 | 7.1 |  |  |  |  |  |  |
| Service personnel | 14 | 7.7 |  |  |  |  |  |  |
| Other | 30 | 16.4 |  |  |  |  |  |  |
| Retirement | 33 | 18.0 |  |  |  |  |  |  |
| ***Education*** |  |  | 6.874 | 0.333 | 5.339 | 0.501 | 2.103 | 0.910 |
| Elementary school and below | 53 | 29.0 |  |  |  |  |  |  |
| High school/secondary school | 65 | 35.5 |  |  |  |  |  |  |
| Post-secondary | 42 | 23.0 |  |  |  |  |  |  |
| College and above | 23 | 12.6 |  |  |  |  |  |  |
| ***Marital status*** |  |  | 0.796 | 0.372 | 6.853 | 0.144 | 6.763 | 0.149 |
| Unmarried | 7 | 3.8 |  |  |  |  |  |  |
| Married | 174 | 95.1 |  |  |  |  |  |  |
| Divorced/Widowed | 2 | 1.1 |  |  |  |  |  |  |
| ***Place of residence*** |  |  | 35.282 | ＜0.001 | 9.793 | 0.134 | 3.712 | 0.716 |
| Urban | 107 | 58.5 |  |  |  |  |  |  |
| County | 42 | 23.0 |  |  |  |  |  |  |
| Town | 20 | 10.9 |  |  |  |  |  |  |
| Rural | 14 | 7.7 |  |  |  |  |  |  |
| ***Residential status*** |  |  | 3.426 | 0.180 | 0.830 | 0.660 | 1.846 | 0.397 |
| Live alone | 8 | 4.4 |  |  |  |  |  |  |
| Live with families | 175 | 95.6 |  |  |  |  |  |  |
| ***Smoking history*** | 62 | 33.9 | 5.774 | 0.056 | 0.451 | 0.798 | 2.944 | 0.229 |
| ***Drinking history*** | 50 | 27.3 | 6.021 | 0.049 | 4.358 | 0.113 | 0.861 | 0.650 |
| ***Gross annual household income* (ten thousand yuan)** |  |  | 13.771 | 0.184 | 11.836 | 0.296 | 16.704 | 0.081 |
| <1 | 5 | 2.7 |  |  |  |  |  |  |
| 1-3 | 17 | 9.3 |  |  |  |  |  |  |
| 4-8 | 65 | 35.5 |  |  |  |  |  |  |
| 9-15 | 76 | 41.5 |  |  |  |  |  |  |
| 16-30 | 13 | 7.1 |  |  |  |  |  |  |
| 31-100 | 7 | 3.8 |  |  |  |  |  |  |
| ***Medical insurance*** |  |  | 3.019 | 0.555 | 8.452 | 0.076 | 5.772 | 0.217 |
| UEBMI | 69 | 37.7 |  |  |  |  |  |  |
| URRBMI | 84 | 45.9 |  |  |  |  |  |  |
| Self-financed | 30 | 16.4 |  |  |  |  |  |  |
| ***Chronic disease*** | 114 | 62.3 | 0.539 | 0.764 | 2.602 | 0.272 | 1.823 | 0.402 |
| ***Stroke type*** |  |  | 13.563 | 0.009 | 7.920 | 0.095 | 11.002 | 0.027 |
| Ischemic stroke | 177 | 96.7 |  |  |  |  |  |  |
| Hemorrhagic stroke | 3 | 1.6 |  |  |  |  |  |  |
| Ischemic combined with hemorrhagic stroke | 3 | 1.6 |  |  |  |  |  |  |
| ***BMI (kg/m^2^)*** |  |  | 1.346 | 0.246 | 0.737 | 0.391 | 4.469 | 0.035 |
| ＜18.5 | 1 | 0.5 |  |  |  |  |  |  |
| 18.5-23.9 | 95 | 51.9 |  |  |  |  |  |  |
| ≥23.9 | 87 | 47.5 |  |  |  |  |  |  |
| ***Rehabilitation period*** |  |  | 40.390 | ＜0.001 | 24.361 | ＜0.001 | 6.982 | 0.030 |
| Initial recovery | 62 | 33.9 |  |  |  |  |  |  |
| Ongoing recovery | 121 | 66.1 |  |  |  |  |  |  |
| ***Physical function recovery (mRS)*** |  |  | 0.711 | 0.701 | 1.630 | 0.443 | 0.397 | 0.820 |
| No to mild disability (0–2) | 171 | 93.4 |  |  |  |  |  |  |
| Moderate to severe disability (>2) | 12 | 6.6 |  |  |  |  |  |  |
| ***Swallowing function (WST)*** |  |  | 7.818 | 0.098 | 1.337 | 0.855 | 1.639 | 0.802 |
| Abnormal | 132 | 72.1 |  |  |  |  |  |  |
| Doubtful | 38 | 20.8 |  |  |  |  |  |  |
| Normal | 13 | 7.1 |  |  |  |  |  |  |
| ***Stroke severity (NIHSS)*** |  |  | 2.204 | 0.332 | 1.170 | 0.557 | 3.251 | 0.197 |
| Mild (≤ 4) | 162 | 88.5 |  |  |  |  |  |  |
| Non-mild (> 4) | 21 | 11.5 |  |  |  |  |  |  |
| ***Activities of daily living (BI)*** |  |  | 0.803 | 0.669 | 0.478 | 0.788 | 6.093 | 0.048 |
| Moderate to severe dependence (≤ 60) | 11 | 6.0 |  |  |  |  |  |  |
| Mild to no dependence (> 60) | 172 | 94.0 |  |  |  |  |  |  |

BMI, Body Mass Index calculated as weight in kilograms divided by height in meters squared, UEBMI Urban Employee Basic Medical Insurance, URRBMI Urban and Rural Resident Basic Medical Insurance, mRS Modified Rankin Scale, WST Water Swallowing Test, NIHSS National Institute of Health Stroke Scale, BI Barthel Index.
