# Supplementary material for: Examining the association between diet-related situational factor and dietary behavior: an observational study of diet-related situational factors in stroke patients during rehabilitation
Source: Front Nutr. 2025 Nov 12;12:1696883. doi: 10.3389/fnut.2025.1696883 (PMC12648219; doi:10.3389/fnut.2025.1696883)
Supplement: Supplementary file 4 [file Table_4.docx]

| **Table4-1** The univariate model of the effects of different meal location on energy intake (n, %) | | | | | | | |
| --- | --- | --- | --- | --- | --- | --- | --- |
| Type of meal | Energy intake | Home | School/Unit | Public Enclosed Place | Public Open Place | ***χ*^2^** | *P* |
|  |  | 496(91.3) | 1(0.2) | 26(4.8) | 21(3.9) |  |  |
| ***Breakfast*** | ***Insufficient*** | 240(48.4) | 0(0.00) | 7(26.9) | 5(23.8) | 16.203 | ＜0.001* |
|  | ***Qualified*** | 188(37.9) | 0(0.00) | 6(23.1) | 11(52.4) |  |  |
|  | ***Excessive*** | 68(13.7) | 1(100.00) | 13(50.00) | 5(23.8) |  |  |
|  |  | 452(82.5) | 6(1.1) | 83(15.1) | 6(1.1) |  |  |
| ***Lunch*** | ***Insufficient*** | 141(31.2) | 1(16.7) | 6(7.2) | 0(0.00) | 32.396 | ＜0.001* |
|  | ***Qualified*** | 188(41.6) | 4(66.7) | 50(60.2) | 2(33.0) |  |  |
|  | ***Excessive*** | 123(27.2) | 1(16.7) | 27(32.5) | 4(66.7) |  |  |
|  |  | 469(85.4) | 5(0.9) | 72(13.1) | 3(0.5) |  |  |
| ***Dinner*** | ***Insufficient*** | 209(44.6) | 1(20.00) | 21(29.2) | 1(33.3) | 3.524 | 0.060 |
|  | ***Qualified*** | 179(38.2) | 1(20.00) | 39(54.2) | 1(33.3) |  |  |
|  | ***Excessive*** | 81(17.3) | 3(60.00) | 12(16.7) | 1(33.3) |  |  |

| **Table4-2** Pairwise Comparison of Breakfast Energy Intake Among Different Meal Location (Holm and BH Corrections) | | | |
| --- | --- | --- | --- |
| Comparison | ***Raw P*** | ***Adjusted P (Holm)*** | ***Adjusted p (BH)*** |
| ***Home vs School/Unit*** | 0.139 | 0.416 | 0.208 |
| ***Home vs Public Enclosed Place*** | <0.001* | <0.001* | <0.001* |
| ***Home vs Public Open Place*** | 0.057 | 0.286 | 0.172 |
| ***School/Unit vs Public Enclosed Place*** | 1.000 | 1.000 | 1.000 |
| ***School/Unit vs Public Open Place*** | 0.500 | 1.000 | 0.600 |
| ***Public Enclosed Place vs Public Open Place*** | 0.100 | 0.400 | 0.200 |

| **Table4-3** Pairwise Comparison of Lunch Energy Intake Among Different Meal Location (Holm and BH Corrections) | | | |
| --- | --- | --- | --- |
| Comparison | ***Raw P*** | ***Adjusted P (Holm)*** | ***Adjusted p (BH)*** |
| ***Home vs School/Unit*** | <0.001* | 0.970 | 0.584 |
| ***Home vs Public Enclosed Place*** | <0.001* | <0.001* | <0.001* |
| ***Home vs Public Open Place*** | <0.001* | 0.348 | 0.209 |
| ***School/Unit vs Public Enclosed Place*** | <0.001* | 0.970 | 0.467 |
| ***School/Unit vs Public Open Place*** | <0.001* | 0.970 | 0.422 |
| ***Public Enclosed Place vs Public Open Place*** | <0.001* | 0.970 | 0.422 |
