# Supplementary material for: Examining the association between diet-related situational factor and dietary behavior: an observational study of diet-related situational factors in stroke patients during rehabilitation
Source: Front Nutr. 2025 Nov 12;12:1696883. doi: 10.3389/fnut.2025.1696883 (PMC12648219; doi:10.3389/fnut.2025.1696883)
Supplement: Supplementary file 5 [file Table_5.docx]

| **Table5-1** The univariate model of the effects of different meal companions on energy intake (n, %) | | | | | | | |
| --- | --- | --- | --- | --- | --- | --- | --- |
| Type of meal | Energy intake | Alone | Family | Colleagues | Friends | ***χ*^2^** | *P* |
|  |  | 83(15.3) | 429(79.0) | 1(0.2) | 31(5.7) |  |  |
| ***Breakfast*** | ***Insufficient*** | 48(57.8) | 188(43.8) | 0(0.00) | 16(51.6) | 0.319 | 0.572 |
|  | ***Qualified*** | 21(25.3) | 173(40.3) | 1(100.00) | 10(32.3) |  |  |
|  | ***Excessive*** | 14(16.9) | 68(15.9) | 0(0.00) | 5(16.1) |  |  |
|  |  | 36(6.6) | 435(79.4) | 1(0.2) | 75(13.7) |  |  |
| ***Lunch*** | ***Insufficient*** | 19(52.8) | 116(26.7) | 0(0.00) | 13(17.3) | 6.477 | 0.011 |
|  | ***Qualified*** | 9(25.0) | 197(45.3) | 1(100.00) | 37(49.3) |  |  |
|  | ***Excessive*** | 8(22.2) | 122(28.0) | 0(00.00) | 25(33.3) |  |  |
|  |  | 40(7.3) | 425(77.4) | 6(1.1) | 78(14.2) |  |  |
| ***Dinner*** | ***Insufficient*** | 25(62.5) | 184(43.3) | 2(33.3) | 21(26.9) | 15.370 | 0.018 |
|  | ***Qualified*** | 11(27.5) | 165(38.8) | 3(50.00) | 41(52.6) |  |  |
|  | ***Excessive*** | 4(10.0) | 76(17.9) | 1(16.7) | 16(20.5) |  |  |

| **Table5-2** Pairwise Comparison of Lunch Energy Intake Among Different Meal Companions (Holm and BH Corrections) | | | |
| --- | --- | --- | --- |
| Comparison | ***Raw P*** | ***Adjusted P (Holm)*** | ***Adjusted p (BH)*** |
| ***Alone vs Family*** | 0.005 | 0.025 | 0.015 |
| ***Alone vs Colleagues*** | 0.486 | 1.000 | 0.730 |
| ***Alone vs Friends*** | <0.001* | 0.004 | 0.004 |
| ***Family vs Colleagues*** | 1.000 | 1.000 | 1.000 |
| ***Family vs Friends*** | 0.207 | 0.829 | 0.415 |
| ***Colleagues vs Friends*** | 1.000 | 1.000 | 1.000 |

| **Table5-3** Pairwise Comparison of Dinner Energy Intake Among Different Meal Companions (Holm and BH Corrections) | | | |
| --- | --- | --- | --- |
| Comparison | ***Raw P*** | ***Adjusted P (Holm)*** | ***Adjusted p (BH)*** |
| ***Alone vs Family*** | 0.071 | 0.286 | 0.143 |
| ***Alone vs Colleagues*** | 0.322 | 0.966 | 0.483 |
| ***Alone vs Friends*** | <0.001* | 0.006 | 0.006 |
| ***Family vs Colleagues*** | 0.867 | 1.000 | 1.000 |
| ***Family vs Friends*** | 0.019 | 0.096 | 0.058 |
| ***Colleagues vs Friends*** | 1.000 | 1.000 | 1.000 |
