# Supplementary material for: Examining the association between diet-related situational factor and dietary behavior: an observational study of diet-related situational factors in stroke patients during rehabilitation
Source: Front Nutr. 2025 Nov 12;12:1696883. doi: 10.3389/fnut.2025.1696883 (PMC12648219; doi:10.3389/fnut.2025.1696883)
Supplement: Supplementary file 6 [file Table_6.docx]

| **Table6-1** The univariate model of the effects of different intention to eat on energy intake (n, %) | | | | | | | | | | |
| --- | --- | --- | --- | --- | --- | --- | --- | --- | --- | --- |
| Type of meal | Energy intake | Habit of eating at this time | Feeling hungry/thirsty | Avoiding hunger/thirst later | Wanting to taste good food | Feeling bored | Feeling stressed | Feeling annoyed | ***χ*^2^** | *P* |
|  |  | 90(16.6) | 327(60.2) | 77(14.2) | 29(5.3) | 11(2.0) | 5(0.9) | 5(0.9) |  |  |
| ***Breakfast*** | ***Insufficient*** | 37(41.1) | 155(47.4) | 34(44.2) | 17(58.6) | 3(27.3) | 3(60.00) | 3(60.00) | 0.725 | 0.394 |
|  | ***Qualified*** | 37(41.1) | 119(36.4) | 33(42.9) | 7(24.1) | 6(54.5) | 2(40.00) | 1(20.00) |  |  |
|  | ***Excessive*** | 16(14.4) | 53(16.2) | 10(13.0) | 5(17.2) | 2(18.2) | 0(0.00) | 1(20.00) |  |  |
|  |  | 53(9.7) | 347(63.3) | 93(17.0) | 37(6.7) | 10(6.6) | 4(0.7) | 3(0.5) |  |  |
| ***Lunch*** | ***Insufficient*** | 12(22.6) | 82(23.6) | 28(30.1) | 17(45.9) | 5(50.00) | 2(50.0) | 2(66.7) | 5.644 | 0.018 |
|  | ***Qualified*** | 31(58.5) | 161(46.4) | 33(35.5) | 15(40.5) | 3(30.00) | 1(25.00) | 0(0.00) |  |  |
|  | ***Excessive*** | 10(18.9) | 104(30.0) | 32(34.4) | 5(13.5) | 2(20.00) | 1(25.0) | 1(33.3) |  |  |
|  |  | 73(13.3) | 284(51.7) | 101(18.4) | 63(11.5) | 14(2.6) | 9(1.6) | 5(0.9) |  |  |
| ***Dinner*** | ***Insufficient*** | 30(41.1) | 128(45.1) | 37(36.6) | 25(39.7) | 5(35.7) | 5(55.6) | 2(40.00) | 0.047 | 0.829 |
|  | ***Qualified*** | 33(45.2) | 103(36.3) | 45(44.6) | 26(41.3) | 7(50.00) | 3(33.3) | 3(60.00) |  |  |
|  | ***Excessive*** | 10(13.7) | 53(18.7) | 19(18.8) | 12(19.0) | 2(14.3) | 1(11.1) | 0(0.00) |  |  |

| **Table5-2** Pairwise Comparison of Lunch Energy Intake Among Different Intention to Eat (Holm and BH Corrections) | | | |
| --- | --- | --- | --- |
| Comparison | ***Raw P*** | ***Adjusted P (Holm)*** | ***Adjusted p (BH)*** |
| ***Habit of eating at this time vs Feeling hungry/thirsty*** | 0.186 | 1.000 | 0.435 |
| ***Habit of eating at this time vs Avoiding hunger/thirst later*** | 0.022 | 0.448 | 0.235 |
| ***Habit of eating at this time vs Wanting to taste good food*** | 0.072 | 1.000 | 0.321 |
| ***Habit of eating at this time vs Feeling bored*** | 0.150 | 1.000 | 0.406 |
| ***Habit of eating at this time vs Feeling stressed*** | 0.287 | 1.000 | 0.535 |
| ***Habit of eating at this time vs Feeling annoyed*** | 0.083 | 1.000 | 0.321 |
| ***Feeling hungry/thirsty vs Avoiding hunger/thirst later*** | 0.155 | 1.000 | 0.406 |
| ***Feeling hungry/thirsty vs Wanting to taste good food*** | 0.010 | 0.208 | 0.208 |
| ***Feeling hungry/thirsty vs Feeling bored*** | 0.234 | 1.000 | 0.492 |
| ***Feeling hungry/thirsty vs Feeling stressed*** | 0.463 | 1.000 | 0.649 |
| ***Feeling hungry/thirsty vs Feeling annoyed*** | 0.092 | 1.000 | 0.321 |
| ***Avoiding hunger/thirst later vs Wanting to taste good food*** | 0.036 | 0.691 | 0.255 |
| ***Avoiding hunger/thirst later vs Feeling bored*** | 0.414 | 1.000 | 0.621 |
| ***Avoiding hunger/thirst later vs Feeling stressed*** | 0.684 | 1.000 | 0.898 |
| ***Avoiding hunger/thirst later vs Feeling annoyed*** | 0.306 | 1.000 | 0.535 |
| ***Wanting to taste good food vs Feeling bored*** | 0.800 | 1.000 | 0.934 |
| ***Wanting to taste good food vs Feeling stressed*** | 0.797 | 1.000 | 0.934 |
| ***Wanting to taste good food vs Feeling annoyed*** | 0.365 | 1.000 | 0.590 |
| ***Feeling bored vs Feeling stressed*** | 1.000 | 1.000 | 1.000 |
| ***Feeling bored vs Feeling annoyed*** | 1.000 | 1.000 | 1.000 |
| ***Feeling stressed vs Feeling annoyed*** | 1.000 | 1.000 | 1.000 |
