# Supplementary material for: Examining the association between diet-related situational factor and dietary behavior: an observational study of diet-related situational factors in stroke patients during rehabilitation
Source: Front Nutr. 2025 Nov 12;12:1696883. doi: 10.3389/fnut.2025.1696883 (PMC12648219; doi:10.3389/fnut.2025.1696883)
Supplement: Supplementary file 7 [file Table_7.docx]

| **Table7** The univariate model of the effects of different situational factors on energy intake (n, %) | | | | | | | | | | | | | |
| --- | --- | --- | --- | --- | --- | --- | --- | --- | --- | --- | --- | --- | --- |
| Type of meal | Energy intake | Degree of help needed with meals | | ***χ*^2^** | *P* | Degree of satisfaction with meals | | ***χ*^2^** | *P* | Degree of anxiety/depression at meals | | ***χ*^2^** | *P* |
|  |  | <3 | ≥3 |  |  | <3 | ≥3 |  |  | <3 | ≥3 |  |  |
| ***Breakfast*** |  | 204(37.6) | 319(58.7) |  |  | 19(3.5) | 524(96.5) |  |  | 453(83.4) | 91(16.8) |  |  |
|  | ***Insufficient*** | 88(39.1) | 164(51.4) | 25.703 | ＜0.001 | 11(57.9) | 241(45.9) | 1.151 | 0.562 | 212(46.8) | 40(44.0) | 4.757 | 0.093 |
|  | ***Qualified*** | 80(35.6) | 125(39.2) |  |  | 6(31.6) | 199(37.9) |  |  | 163(36.0) | 42(34.3) |  |  |
|  | ***Excessive*** | 36(25.3) | 30(9.4) |  |  | 2(10.5) | 84(16.2) |  |  | 78(17.2) | 9(14.6) |  |  |
| ***Lunch*** |  | 219(40.0) | 328(60.0) |  |  | 27(4.9) | 520(95.1) |  |  | 454(83.0) | 93(17.0) |  |  |
|  | ***Insufficient*** | 46(21.0) | 102(31.1) | 29.611 | ＜0.001 | 5(18.5) | 143(27.5) | 1.095 | 0.578 | 119(26.2) | 29(31.2) | 5.588 | 0.061 |
|  | ***Qualified*** | 83(37.9) | 161(49.1) |  |  | 13(48.1) | 231(44.4) |  |  | 197(43.4) | 47(50.5) |  |  |
|  | ***Excessive*** | 90(41.1) | 65(19.8) |  |  | 9(33.3) | 146(28.1) |  |  | 138(30.4) | 17(18.3) |  |  |
| ***Dinner*** |  | 252(45.9) | 297(54.1) |  |  | 39(7.1) | 510(92.9) |  |  | 437(79.6) | 112(20.4) |  |  |
|  | ***Insufficient*** | 95(37.7) | 137(46.1) | 4.200 | 0.122 | 27(69.2) | 205(40.2) | 12.541 | 0.002 | 177(40.5) | 55(49.1) | 4.509 | 0.105 |
|  | ***Qualified*** | 107(42.5) | 113(38.0) |  |  | 8(20.5) | 212(41.6) |  |  | 176(40.3) | 44(39.3) |  |  |
|  | ***Excessive*** | 50(19.8) | 47(15.8) |  |  | 4(10.3) | 93(18.2) |  |  | 84(19.2) | 13(11.6) |  |  |
