# Supplementary material for: Examining the association between diet-related situational factor and dietary behavior: an observational study of diet-related situational factors in stroke patients during rehabilitation
Source: Front Nutr. 2025 Nov 12;12:1696883. doi: 10.3389/fnut.2025.1696883 (PMC12648219; doi:10.3389/fnut.2025.1696883)
Supplement: Supplementary file 8 [file Table_8.docx]

| **Table 8** The univariate model of the effects of different situational factors on energy intake (n, %) | | | | | | | | | | | | | |
| --- | --- | --- | --- | --- | --- | --- | --- | --- | --- | --- | --- | --- | --- |
| Type of meal | Energy intake | Degree of quietness of the environment during the meal | | ***χ*^2^** | *P* | Ability to cook independently | | ***χ*^2^** | *P* | Ability to shop for groceries independently | | ***χ*^2^** | *P* |
|  |  | <3 | ≥3 |  |  | <3 | ≥3 |  |  | <3 | ≥3 |  |  |
| ***Breakfast*** |  | 227(41.7) | 317(58.3) |  |  | 229(42.1) | 315(57.9) |  |  | 228(41.9) | 316(58.1) |  |  |
|  | ***Insufficient*** | 89(39.2) | 163(51.4) | 22.850 | ＜0.001 | 90(39.3) | 162(51.4) | 24.159 | ＜0.001 | 89(44.2) | 163(44.2) | 24.721 | ＜0.001 |
|  | ***Qualified*** | 82(36.1) | 123(38.8) |  |  | 82(35.8) | 123(39.0) |  |  | 82(42.9) | 123(42.9) |  |  |
|  | ***Excessive*** | 56(24.7) | 31(9.8) |  |  | 57(24.9) | 30(9.5) |  |  | 57(13.0) | 30(13.0) |  |  |
| ***Lunch*** |  | 218(39.9) | 329(60.1) |  |  | 217(39.7) | 330(60.3) |  |  | 217(39.7) | 330(60.3) |  |  |
|  | ***Insufficient*** | 44(20.2) | 104(31.6) | 30.771 | ＜0.001 | 44(20.3) | 104(31.5) | 31.282 | ＜0.001 | 44(20.3) | 104(31.5) | 29.317 | ＜0.001 |
|  | ***Qualified*** | 84(38.5) | 160(48.6) |  |  | 83(38.2) | 161(48.8) |  |  | 84(38.7) | 160(48.5) |  |  |
|  | ***Excessive*** | 90(41.3) | 65(19.8) |  |  | 90(41.5) | 65(19.7) |  |  | 89(41.0) | 66(20.0) |  |  |
| ***Dinner*** |  | 235(42.8) | 314(57.2) |  |  | 239(43.5) | 310(56.5) |  |  | 238(43.4) | 311(56.6) |  |  |
|  | ***Insufficient*** | 93(39.6) | 139(44.3) | 12.352 | 0.002 | 94(39.3) | 138(44.5) | 16.252 | ＜0.001 | 93(39.1) | 139(44.7) | 14.693 | 0.001 |
|  | ***Qualified*** | 85(36.2) | 135(43.0) |  |  | 85(35.6) | 135(43.5) |  |  | 86(36.1) | 134(43.1) |  |  |
|  | ***Excessive*** | 57(24.3) | 40(12.7) |  |  | 60(25.1) | 37(11.9) |  |  | 59(24.8) | 38(12.2) |  |  |
