# Supplementary material for: Examining the association between diet-related situational factor and dietary behavior: an observational study of diet-related situational factors in stroke patients during rehabilitation
Source: Front Nutr. 2025 Nov 12;12:1696883. doi: 10.3389/fnut.2025.1696883 (PMC12648219; doi:10.3389/fnut.2025.1696883)
Supplement: Supplementary file 9 [file Table_9.docx]

| **Table9** Multinomial logistic regression model of the effects of different situational factor on breakfast energy intake | | | | | | | | |
| --- | --- | --- | --- | --- | --- | --- | --- | --- |
| Characteristic | β | S.E. | OR | 95%CI | | | | *P* |
|  |  |  |  | Lower limit | Upper limit | | |  |
| ***Insufficient energy intake compared with qualified energy intake*** | | | | | | | | |
| Constant | -4.663 | 1.488 | - | - | | | - | 0.002 |
| Gender | -0.754 | 0.256 | 0.471 | 0.285 | | | -0.777 | 0.003 |
| Age | 0.581 | 0.429 | 1.788 | 4.148 | | | 4.148 | 0.176 |
| Occupation | 0.029 | 0.066 | 1.030 | 0.905 | | | 1.171 | 0.657 |
| Place of residence | -0.024 | 0.137 | 0.976 | 0.747 | | | 1.275 | 0.858 |
| Drinking history | 0.209 | 0.245 | 1.232 | 0.763 | | | 1.991 | 0.394 |
| Stroke type | 1.976 | 0.980 | 7.216 | 1.057 | | | 49.270 | 0.044 |
| Rehabilitation period | 1.154 | 0.302 | 3.170 | 1.754 | | | 5.728 | ＜0.001 |
| Degree of help needed with meals | 0.209 | 0.273 | 1.233 | 0.721 | | | 2.107 | 0.444 |
| Degree of quietness of the environment during the meal | -0.150 | 0.206 | 0.861 | 0.575 | | | 1.288 | 0.465 |
| Ability to cook independently | -0.113 | 0.204 | 0.893 | 0.599- | | 1.332 | | 0.579 |
| Ability to shop for groceries independently | 0.171 | 0.204 | 0.843 | 0.565 | | 1.256 | | 0.401 |
| Meal location |  |  |  |  | |  | |  |
| Public Open Place | Reference |  |  |  | |  | |  |
| Home | 0.843 | 0.561 | 2.323 | 0.773 | | 6.980 | | 0.133 |
| School/Unit | -0.271 | 0.000 | 0.763 | 0.763 | | 0.763 | | - |
| Public Enclosed Place | 0.720 | 0.796 | 2.055 | 0.432 | | 9.781 | | 0.366 |
| ***Excessive energy intake compared with qualified energy intake*** | | | | | | | | |
| Constant | -5.715 | 1.774 | - | - | - | | | 0.001 |
| Gender | 0.640 | 0.383 | 1.897 | 0.895 | 4.019 | | | 0.095 |
| Age | 1.010 | 0.484 | 2.747 | 1.063 | 7.097 | | | 0.037 |
| Occupation | 0.730 | 0.096 | 1.075 | 0.890 | 1.298 | | | 0.451 |
| Place of residence | 0.228 | 0.164 | 1.257 | 0.912 | 1.732 | | | 0.163 |
| Drinking history | -0.949 | 0.368 | 0.387 | 0.188 | 0.796 | | | 0.010 |
| Stroke type | 2.279 | 1.052 | 9.768 | 1.241 | 76.853 | | | 0.030 |
| Rehabilitation period | -0.201 | 0.422 | 0.818 | 0.358 | 1.870 | | | 0.634 |
| Degree of help needed with meals | 0.484 | 0.425 | 1.622 | 0.705 | 3.732 | | | 0.255 |
| Degree of quietness of the environment during the meal | 0.992 | 0.303 | 2.696 | 1.489 | 4.884 | | | 0.001 |
| Ability to cook independently | 0.995 | 0.301 | 2.704 | 1.500 | 4.876 | | | 0.001 |
| Ability to shop for groceries independently | 1.227 | 0.307 | 3.411 | 1.867 | 6.232 | | | ＜0.001 |
| Meal location |  |  |  |  |  | | |  |
| Public Open Place | Reference |  |  |  |  | | |  |
| Home | -0.426 | 0.643 | 0.653 | 0.185 | 2.304 | | | 0.508 |
| School/Unit | 16.315 | 0.000 | - | - | - | | | - |
| Public Enclosed Place | 1.773 | 0.841 | 2.055 | 1.132 | 30.641 | | | 0.035 |
