# Supplementary material for: Examining the association between diet-related situational factor and dietary behavior: an observational study of diet-related situational factors in stroke patients during rehabilitation
Source: Front Nutr. 2025 Nov 12;12:1696883. doi: 10.3389/fnut.2025.1696883 (PMC12648219; doi:10.3389/fnut.2025.1696883)
Supplement: Supplementary file 10 [file Table_10.docx]

| **Table10** Multinomial logistic regression model of the effects of different situational factor on lunch energy intake | | | | | | | | |
| --- | --- | --- | --- | --- | --- | --- | --- | --- |
| Characteristic | β | S.E. | OR | 95%CI | | | | *P* |
|  |  |  |  | Lower limit | Upper limit | | |  |
| ***Insufficient energy intake compared with qualified energy intake*** | | | | | | | | |
| Constant | 1.850 | 7212.440 | - | - | | |  | 1.000 |
| Gender | -0.757 | 0.286 | 0.469 | 0.268 | | | 0.822 | 0.008 |
| Age | -0.499 | 0.481 | 0.607 | 0.237 | | | 1.557 | 0.299 |
| Occupation | 0.004 | 0.073 | 1.004 | 0.869 | | | 1.159 | 0.960 |
| Rehabilitation period | 0.146 | 0.337 | 1.157 | 0.597 | | | 2.242 | 0.666 |
| Degree of help needed with meals | -0.206 | 0.311 | 0.814 | 0.442 | | | 1.498 | 0.508 |
| Degree of quietness of the environment during the meal | -0.240 | 0.243 | 0.787 | 0.488 | | | 1.267 | 0.324 |
| Ability to cook independently | -0.267 | 0.244 | 0.766 | 0.475 | | | 1.235 | 0.273 |
| Ability to shop for groceries independently | -0.272 | 0.244 | 0.762 | 0.472 | | | 1.228 | 0.264 |
| Meal companions |  |  |  |  | | |  |  |
| Friends | Reference |  |  |  | | |  |  |
| Alone | 1.883 | 0.568 | 6.573 | 2.161 | | | 19.994 | 0.001 |
| Family | 0.322 | 0.383 | 1.380 | 0.651 | | | 2.925 | 0.401 |
| ***Excessive energy intake compared with qualified energy intake*** | | | | | | | | |
| Constant | 17.862 | 5906.322 | - | - | | |  | 0.998 |
| Gender | 0.089 | 0.269 | 1.093 | 0.646 | | | 1.851 | 0.740 |
| Age | -0.128 | 0.408 | 0.880 | 0.396 | | | 1.956 | 0.754 |
| Occupation | 0.054 | 0.076 | 1.056 | 0.909 | | | 1.227 | 0.476 |
| Rehabilitation period | -0.624 | 0.323 | 0.536 | 0.285 | | | 1.008 | 0.053 |
| Degree of help needed with meals | 0.705 | 0.317 | 2.024 | 1.088 | | | 3.766 | 0.026 |
| Degree of quietness of the environment during the meal | 0.983 | 0.236 | 2.672 | 1.682 | | | 4.245 | ＜0.001 |
| Ability to cook independently | 0.987 | 0.237 | 2.682 | 1.684 | | 4.272 | | ＜0.001 |
| Ability to shop for groceries independently | 0.951 | 0.235 | 2.589 | 1.633 | | 4.103 | | ＜0.001 |
| Meal location |  |  |  |  | |  | |  |
| Public Open Place | Reference |  |  |  | |  | |  |
| Home | -1.571 | 1.066 | 0.208 | 0.026 | | 1.680 | | 0.141 |
| School/Unit | -2.601 | 1.637 | 0.074 | 0.003 | | 1.837 | | 0.112 |
| Public Enclosed Place | -1.424 | 1.090 | 0.241 | 0.028 | | 2.036 | | 0.191 |
| Meal companions |  |  |  |  | |  | |  |
| Friends | Reference |  |  |  | |  | |  |
| Alone | 0.014 | 0.625 | 1.014 | 0.298 | | 3.455 | | 0.982 |
| Family | -0.246 | 0.336 | 0.782 | 0.404 | | 1.511 | | 0.464 |
