# Supplementary material for: Examining the association between diet-related situational factor and dietary behavior: an observational study of diet-related situational factors in stroke patients during rehabilitation
Source: Front Nutr. 2025 Nov 12;12:1696883. doi: 10.3389/fnut.2025.1696883 (PMC12648219; doi:10.3389/fnut.2025.1696883)
Supplement: Supplementary file 11 [file Table_11.docx]

| **Table11** Multinomial logistic regression model of the effects of different situational factor on dinner energy intake | | | | | | |
| --- | --- | --- | --- | --- | --- | --- |
| Characteristic | β | S.E. | OR | 95%CI | | *P* |
|  |  |  |  | Lower limit | Upper limit |  |
| ***Insufficient energy intake compared with qualified energy intake*** | | | | | | |
| Constant | -1.440 | 1.166 | - | - |  | 0.217 |
| Gender | -0.575 | 0.233 | 0.563 | 0.357 | 0.888 | 0.013 |
| Stroke type | -0.187 | 0.324 | 0.829 | 0.440 | 1.564 | 0.563 |
| BMI | 0.234 | 0.203 | 1.263 | 0.849 | 1.879 | 0.248 |
| Rehabilitation period | 0.039 | 0.218 | 1.040 | 0.679 | 1.594 | 0.856 |
| Activities of daily living (BI) | 0.442 | 0.397 | 1.556 | 0.714 | 3.388 | 0.266 |
| Meal companions |  |  |  |  |  |  |
| Friends | Reference |  |  |  |  |  |
| Alone | 1.575 | 0.471 | 4.833 | 1.921 | 12.159 | 0.001 |
| Family | 0.815 | 0.298 | 2.259 | 1.259 | 4.053 | 0.006 |
| Colleagues | 0.442 | 0.981 | 1.556 | 0.227 | 10.637 | 0.652 |
| Degree of satisfaction with meals | 1.186 | 0.421 | 3.274 | 1.434 | 7.477 | 0.005 |
| Degree of quietness of the environment during the meal | -0.027 | 0.201 | 0.974 | 0.657 | 1.444 | 0.895 |
| Ability to cook independently | 0.122 | 0.201 | 1.130 | 0.763 | 1.674 | 0.542 |
| Ability to shop for groceries independently | 0.004 | 0.203 | 1.004 | 0.674 | 1.494 | 0.984 |
| ***Excessive energy intake compared with qualified energy intake*** | | | | | | |
| Constant | 11.263 | 2.352 | - | - | - | ＜0.001 |
| Gender | 0.167 | 0.286 | 1.182 | 0.674 | 2.071 | 0.560 |
| BMI | -0.272 | 0.262 | 0.762 | 0.455 | 1.274 | 0.300 |
| Rehabilitation period | -1.060 | 0.282 | 0.347 | 0.199 | 0.602 | ＜0.001 |
| Activities of daily living (BI) | 2.429 | 1.064 | 11.343 | 1.409 | 91.301 | 0.022 |
| Meal companions |  |  |  |  |  |  |
| Friends | Reference |  |  |  |  |  |
| Alone | -0.876 | 0.709 | 0.417 | 0.104 | 1.671 | 0.217 |
| Family | 0.003 | 0.359 | 1.003 | 0.496 | 2.028 | 0.994 |
| Colleagues | -0.408 | 1.324 | 0.665 | 0.050 | 8.912 | 0.758 |
| Degree of satisfaction with meals | 0.247 | 0.665 | 1.280 | 0.348 | 4.712 | 0.710 |
| Degree of quietness of the environment during the meal | 0.955 | 0.269 | 2.598 | 1.533 | 4.401 | ＜0.001 |
| Ability to cook independently | 0.967 | 0.270 | 2.631 | 1.551 | 4.463 | ＜0.001 |
| Ability to shop for groceries independently | 1.065 | 0.271 | 2.902 | 1.707 | 4.934 | ＜0.001 |
