# Supplementary figures and images for: Examining the association between diet-related situational factor and dietary behavior: an observational study of diet-related situational factors in stroke patients during rehabilitation
Source: Front Nutr. 2025 Nov 12;12:1696883. doi: 10.3389/fnut.2025.1696883 (PMC12648219; doi:10.3389/fnut.2025.1696883)

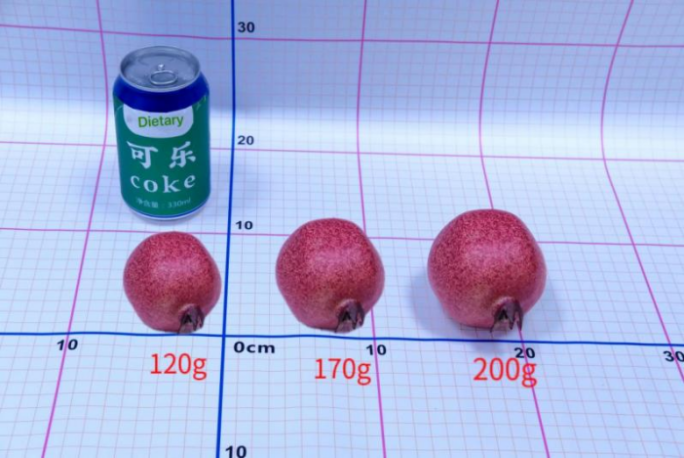

Supplement: Supplementary Figure 1 — Food atlas. [file Image_1.jpeg]

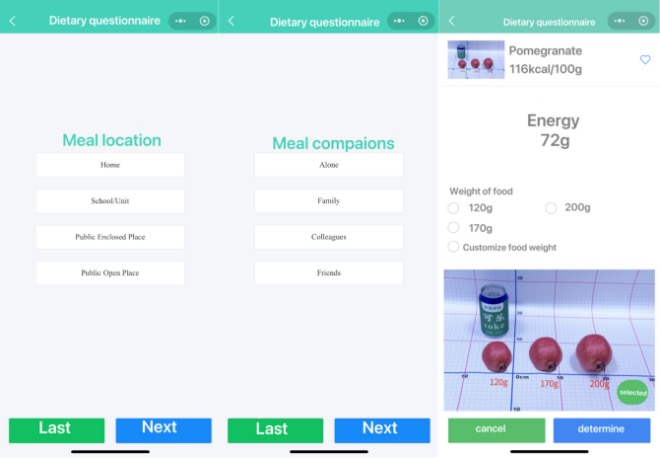

Supplement: Supplementary Figure 2 — Information platform. [file Image_2.jpeg]

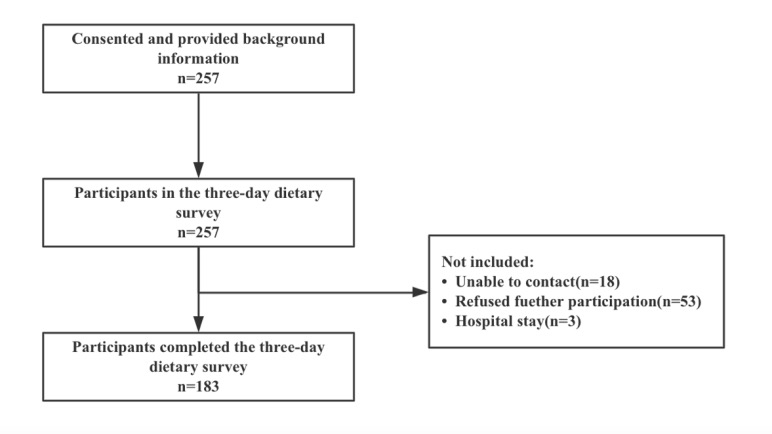

Supplement: Supplementary Figure 3 — Recruitment flowchart. [file Image_3.jpeg]
